# Enhanced efficacy of combination therapy with adeno-associated virus-delivered pigment epithelium-derived factor and cisplatin in a mouse model of Lewis lung carcinoma

**DOI:** 10.3892/mmr.2014.2117

**Published:** 2014-04-04

**Authors:** SHA-SHA HE, QIN-JIE WU, CHANG YANG GONG, SHUN-TAO LUO, SHUANG ZHANG, MENG LI, LIAN LU, YU-QUAN WEI, LI YANG

**Affiliations:** State Key Laboratory of Biotherapy, West China Hospital, Sichuan University, Chengdu, Sichuan 610041, P.R. China

**Keywords:** adeno-associated virus-pigment epithelium-derived factor, cisplatin, combination, tumor, apoptosis, angiogenesis

## Abstract

Pigment epithelium-derived factor (PEDF) is a potent inhibitor of angiogenesis, and the antitumor effect of adeno-associated virus (AAV)-mediated PEDF expression has been demonstrated in a range of animal models. The combined treatment of low-dose chemotherapy and gene therapy inhibits the growth of solid tumors more effectively than current traditional therapies or gene therapy alone. In the present study, the effect of treatment with an AAV2 vector harboring the human PEDF (hPEDF) gene in combination with low-dose cisplatin on the growth of Lewis lung carcinoma (LLC) in mice was assessed. LLC cells were infected with AAV-enhanced green fluorescent protein (EGFP) in the presence or absence of cisplatin, and then the effect of cisplatin on AAV-mediated gene expression was evaluated by image and flow cytometric analysis. Tumor growth, survival time, vascular endothelial growth factor (VEGF) expression, microvessel density (MVD) and apoptotic index were analyzed in C57BL/6 mice treated with AAV-hPEDF, cisplatin or cisplatin plus AAV-hPEDF. The results of the present study provide evidence that cisplatin treatment is able to enhance AAV-mediated gene expression in LLC cells. In addition, the combined treatment of cisplatin plus AAV-hPEDF markedly prolonged the survival time of the mice and inhibited tumor growth, resulting in significant suppression of tumor angiogenesis and induction of tumor apoptosis *in vivo*, and also protected against cisplatin-related toxicity. These findings suggest that combination of AAV-hPEDF and cisplatin has potential as a novel therapeutic strategy for lung cancer.

## Introduction

Lung cancer is one of the leading causes of cancer-related mortality worldwide. Cisplatin is an alkylating agent, approved as a first-line chemotherapeutic drug for the disease. However, improvement in the efficacy of conventional chemotherapy on cancer is limited due to severe toxic side effects and acquired resistance (1,2). Certain studies have reported that radiation or chemotherapy administered in combination with gene therapy may provide a greater anticancer therapy response with enhanced antitumor effects and reduced toxicity (3,4). Results from animal studies have suggested that a combination of low-dose chemotherapy with gene therapy for treatment of solid tumors results in more effective inhibition of tumor growth than using gene therapy or traditional chemotherapy alone (3,5).

Gene therapy, transferring therapeutic genes to tumor cells using diverse delivery vehicles, is considered to have potential advantages for treating intractable cancers. AAV is a parvovirus with a 4.7 kb single-stranded DNA genome, and has emerged as a potential vector for mediating gene transfer into tumor cells, in that it is non-pathogenic, has a broad host range, is capable of infecting nondividing and dividing cells, can stably integrate into host DNA, is able to establish long-term transgene expression, and presents low immunogenicity (6). These features render AAV a useful alternative to other viral vectors for human gene therapy. To date, AAV vectors have been utilized in numerous preclinical and clinical studies for the treatment of a number of diseases (7–9). Thus, in the present study, low-dose cisplatin was administered in combination with an AAV_2_ vector encoding the human pigment epithelium-derived factor (hPEDF) gene, to investigate the antitumor effect in a mouse model of Lewis lung carcinoma.

PEDF, an endogenously produced 50-kDa glycoprotein, is a member of the serpin family, which is widely expressed throughout the body, including in the brain, eye, liver, bone, heart and lung (10,11). PEDF was first identified as an effective neurotrophic factor produced by cultured human fetal retinal pigment epithelial cells (12). Recently however, more attention has been paid to its antiangiogenic activity, far greater than that of other known endogenous angiostatic molecules, such as endostatin, angiostatin and thrombospondin-1 (13). The antivascular activity of PEDF appears to be associated with two main mechanisms: Activation of Fas-FasL-mediated apoptosis and downregulation of vascular endothelial growth factor (VEGF) expression (14).

The effectiveness of combination treatment of AAV-hPEDF and low-dose cisplatin on lung cancer has, to the best of our knowledge, not been reported. The present study was designed to evaluate the enhanced efficacy of AAV-hPEDF combined with low-dose cisplatin on the growth of established Lewis lung carcinoma in mice.

## Materials and methods

### Cell lines

LLC cell lines were obtained from the American Type Culture Collection (Manassas, VA, USA). The cells were maintained as monolayers in Dulbecco’s modified Eagle’s medium (DMEM) supplemented with 10% fetal bovine serum (FBS). Human umbilical vein endothelial cells (HUVECs) were isolated from umbilical cords by a standard procedure (15) and then grown in Endothelial Basal Medium-2 (Lonza, Basel, Switzerland) supplemented with VEGF, epidermal growth factor, fibroblast growth factor and R3-IGF-1.

### Construction of AAV-hPEDF and AAV-EGFP vectors

AAV-hPEDF and AAV-enhanced green fluorescent protein (EGFP) were constructed as previously described (16). rAAV viral particles were packaged and purified as described previously (17).

### Transduction with AAV-EGFP or AAV-hPEDF, treatment with cisplatin and flow cytometry

LLC cells were grown in 6-well plates to 70–80% confluence, then the cells were treated with serum-free DMEM in the presence or absence of cisplatin (250 ng/ml; Haosen pharmaceutical Co., Ltd, Jiangsu, China). Two hours after cisplatin administration, the cells were transduced by AAV-hPEDF or AAV-EGFP at a multiplicity of infection (MOI) of 1×10^5^ particles per cell in serum-free DMEM, with or without cisplatin treatment. At 2 h post-infection, the medium was changed to complete medium, with or without cisplatin, and the cells were cultured for 72 h.

Cells treated with AAV-EGFP were collected and resuspended in phosphate-buffered saline (PBS) at a concentration of ~10^6^ cells/ml and fixed with ice-cold absolute ethanol overnight. Subsequently, cellular expression of the EGFP transgene was quantitatively assessed using a flow cytometer (BD Biosciences, San Jose, CA, USA).

### Western blot analysis

LLC cells treated with AAV-EGFP, cisplatin, AAV-hPEDF or cisplatin plus AAV-hPEDF were lysed with radio-immunoprecipitation assay solution, and protein concentrations were then determined with a modified Lowry protein assay kit (Thermo Fisher Scientific, Rockford, IL, USA). Proteins (40 mg) from each sample were loaded onto SDS-PAGE gels and then electrotransferred onto a polyvinylidine fluoride membrane, and probed with anti-hPEDF monoclonal antibody (1:1000; R&D Systems, Boston, MA, USA). Blots were incubated for 1 h with horseradish peroxidase (HRP)-conjugated secondary antibody (1:10,000; ZSJQ Biotechnology, Beijing, China). The proteins on the blots were visualized using an enhanced chemoluminescence system (Pierce Biotechnology, Inc., Rockford, IL, USA).

### Tube formation assay

Tube formation assays were performed as previously described (18). HUVECs were seeded into a Matrigel-coated (BD Biosciences, Franklin Lakes, NJ, USA) 96-well plate, and then treated with conditioned media from LLC cells treated with normal saline (NS), AAV-EGFP, cisplatin, AAV-hPEDF or cisplatin plus AAV-hPEDF, respectively. Six hours later, the tubule branches were photographed (Olympus BX53; Olympus, Tokyo, Japan).

### Animal studies

All mouse experiments were approved by the Animal Care and Use Committee of Sichuan University (Chengdu, Sichuan, China).

Male C57BL/6 mice (age, 8 weeks) were obtained from the Experimental Animal Center of Sichuan University, (Sichuan, China). The LLC cells were inoculated subcutaneously into the back right side of the animals. Seven days after tumor cell injection, when the tumor nodule had reached an average size of 4×5 mm, the animals were randomized into five groups. The groups were administered either AAV-hPEDF (2×10^10^ viral genome copies per 50 μl) intratumorally, cisplatin at a dosage of 2 mg/kg intraperitoneally every three days (a total of six doses), or cisplatin plus AAV-hPEDF simultaneously. The remaining two groups of mice were either treated with AAV-EGFP intratumorally and intraperitoneally injected with NS, or were treated with an intratumoral injection of NS. Tumor volume was measured and calculated using the formula: Tumor volume = 0.52 × length × width^2^.

The mice were sacrificed at the end of experiment; solid tumor tissues were then surgically resected, weighed and processed for routine histological analysis and immunohistochemistry.

For the survival studies, another five groups of mice (n=10) were treated as described and the survival time was recorded.

### Immunohistochemistry

#### Tumor microvessel density and human PEDF expression

Prepared frozen sections of tumors were respectively incubated with anti-mouse CD31 antibody (BD Biosciences, Franklin Lakes, NJ, USA) and anti-human PEDF antibody (R&D Systems, Minneapolis, MN, USA) overnight, and subsequently with a fluorescence-conjugated secondary antibody (1:100; Abcam, Cambridge, MA, USA) for 45 min. The CD31-positive vessels and the PEDF-positive reaction were visualized with 3,3′-diaminobenzidine (DAB; ZSJQ Biotechnology).

#### Caspase-3 staining and VEGF staining

The primary anti-caspase-3 (Cell Signaling Technology, Inc., Danvers, MA, USA) and anti-VEGF (R&D Systems) monoclonal antibodies were applied to paraffin sections of tumor tissues at 4°C overnight. After two washes with PBS, the HRP-conjugated secondary antibody was applied at room temperature for 40 min. Subsequently, DAB was used for signal amplification. The VEGF staining intensity was quantified with Carl Zeiss AxioImager microscope Image M1 Software (Carl Zeiss AG, Jena, Germany).

#### In situ transferase-mediated dUTP nick end labelling (TUNEL) assay

Apoptotic cells in tumor tissues from each group were detected by *in situ* TUNEL analysis, following the instructions of an In situ Apoptosis kit (Promega Corporation, Madison, WI, USA). TUNEL-positive cells were quantified using a fluorescence microscope (Carl Zeiss Microimaging Inc., Thornwood, NY, USA).

#### Cell proliferation assay

LLC cells were seeded into a 96-well plate at a density of 1×10^4^ cells per well overnight, and then treated with different doses of cisplatin (Haosen Pharmaceutical Co., Ltd., Lianyungang, Jiangsu, China). After 72 hours of incubation, cell proliferation was measured using an MTT assay.

#### Statistical analysis

Statistical analysis was conducted using SPSS 18.0 software (SPSS, Inc., Chicago, IL, USA). Data are presented as the mean ± standard deviation. The differences among the five groups were evaluated by one-way analysis of variance. Survival data were analyzed using the log-rank test and P<0.05 was considered to indicate a statistically significant difference.

## Results

### Cisplatin enhances AAV-EGFP expression and AAV-hPEDF expression in LLC cells in vitro

Previous studies have indicated that infection with AAV was enhanced by irradiation, UV light and various chemotherapeutic agents (19,20). In the present study, the effect of cisplatin on transgene expression following AAV vector delivery was investigated. MTT assays were performed using different doses of cisplatin to quantify cisplatin-induced cytotoxicity and to determine a sub-toxic dosage that results in <25% cell death in LLC cells (data not shown). It was found that 72 h treatment with cisplatin inhibited cell proliferation in a dose-dependent manner in LLC cells, and that 250 ng/ml cisplatin administered to the cells for 72 h led to 20% inhibition of cell proliferation, indicating that this was an optimal concentration to be used in combination therapy with AAV-hPEDF.

**Figure 1:**
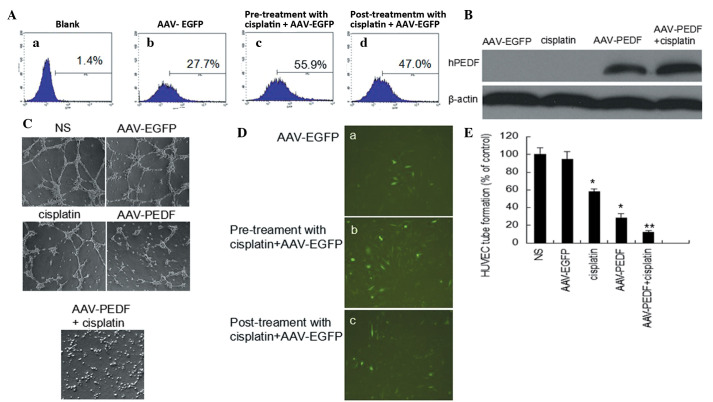
Cisplatin enhanced adeno-associated virus (AAV)-enhanced green fluorescent protein (EGFP) expression and AAV-mediated human pigment epithelium-derived factor (PEDF) expression in the Lewis lung carcinoma (LLC) cell line. (A) The AAV-delivered EGFP expression in LLC cells in the (Aa) absence or (Ab) presence of 250 ng/ml cisplatin for 2 h (Ac) before and (Ad) after AAV-EGFP infection was analyzed by flow cytometry. (B) Western blot assay of LLC cells for human PEDF protein levels. (C) Inhibitory effect of cisplatin plus AAV-hPEDF on human umbilical vein endothelial cell tubule formation (magnification, ×100) and inhibition ratio of tube formation (E). (D) Fluorescence micrographs of LLC cells after the treatments described in A (magnification, ×400). ^*^P<0.05 and ^**^P<0.01 compared with NS or AAV-EGFP.

The ability of cisplatin to induce AAV infection was first investigated using AAV-EGFP (Fig. 1). LLC cells were pre-exposed to 250 ng/ml cisplatin for 2 h prior to AAV-EGFP infection. As revealed in Fig. 1D(a-c), a marked improvement in EGFP expression was observed in the pre-exposed cells compared with cells that received either no treatment, or treatment with cisplatin following AAV-EGFP infection. EGFP expression levels in LLC cells were then analyzed using flow cytometry, and the results demonstrated that pre-treatment with 250 ng/ml cisplatin for 2 h prior to AAV-EGFP transduction led to an EGFP-positive rate in LLC cells of 55.9%, whereas no treatment or treatment with cisplatin following AAV-EGFP infection resulted in EGFP-positive rates of 27.7 and 47%, respectively (Fig. 1A).

LLC cells were treated with 250 ng/ml cisplatin or transduced with AAV-hPEDF in the presence or absence of cisplatin at an MOI of 10^5^ particles per cell. hPEDF transgene expression in LLC cells was analyzed 72 h after infection with AAV-hPEDF, using western blot analysis. In the presence of AAV-hPEDF transduction, hPEDF protein was expressed in LLC cells, and this expression was significantly increased by cisplatin treatment (Fig. 1B).

These results indicate that cisplatin increased AAV-mediated transgene expression in LLC cells.

### Bioactivity of hPEDF produced by AAV-hPEDF-transduced LLCs in vitro

When HUVECs are seeded onto a Matrigel matrix, the cells elongate and form capillary-like cords (21). This assay was used to assess the ability of cells to form capillary-like structures in conditioned media, using LLC cells transfected with NS, AAV-EGFP, cisplatin, AAV-hPEDF or cisplatin plus AAV-hPEDF. Treatment with the conditioned media from AAV-hPEDF markedly inhibited the tube formation; however, the combined treatment of cisplatin and AAV-hPEDF (12±2.3%) resulted in marked inhibition of HUVEC tube formation compared with treatment with either AAV-hPEDF alone (28.5±4.8%) or 250 ng/ml cisplatin alone (58±3.0%) (Fig. 1C and E, P<0.05). The data reveal that the PEDF protein produced by transfected cells was highly bioactive and that treatment with cisplatin and AAV-hPEDF in combination resulted in greater suppression of angiogenesis *in vitro* by inhibiting tube formation.

### Antitumor efficacy of AAV-hPEDF combined with cisplatin in mice with established Lewis lung carcinoma

**Figure 2:**
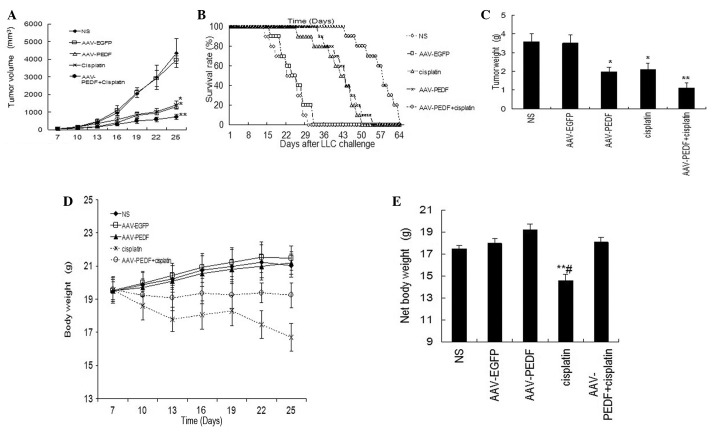
Efficacy of the combination of adeno-associated virus (AAV)-human pigment epithelium-derived factor (hPEDF) and cisplatin on survival, tumor growth and tumor weight. (A) Tumor growth curve. (B) Survival curve. (C) The average Lewis lung carcinoma (LLC) tumor weight of each group. (D) Changes in body weight of mice; there was a significant difference in body weight between the cisplatin group and the control groups. (E) Net body weight was calculated by subtracting the tumor weight from the total body weight.^*^P<0.05 and ^**^P<0.01 compared with normal saline (NS) or AAV-enhanced green fluorescent protein (EGFP), ^#^P<0.05 compared with the combination group.

A mouse LLC tumor model was used to examine the potential inhibitory effect of combination therapy on tumor growth. Treatment was initiated when the tumor nodule reached an average size of 4×5 mm. As shown in Fig. 2A, treatment with either cisplatin or AAV-hPEDF alone resulted in a marked reduction in tumor volume compared with that of the two control groups, NS and AAV-EGFP, during the treatment period (P<0.05). However, treatment with cisplatin plus AAV-hPEDF demonstrated an enhanced inhibitory effect on tumor growth (Fig. 2A, P<0.01). On the 25th day post-inoculation, the mice were sacrificed and solid tumor tissues were surgically resected and weighed (Fig. 2C). The tumor weights of the combination-treated group were markedly lower than those in the groups treated with either cisplatin or AAV-hPEDF alone (Fig. 2C, P<0.05), as well as those in the NS or AAV-EGFP groups (Fig. 2C, P<0.01). Moreover, the group treated with a combination of cisplatin and AAV-hPEDF demonstrated an increased survival rate compared with the control groups (Fig. 2B, P<0.01) or either of the cisplatin- or AAV-hPEDF-treated groups (Fig. 2B, P<0.05).

**Figure 3:**
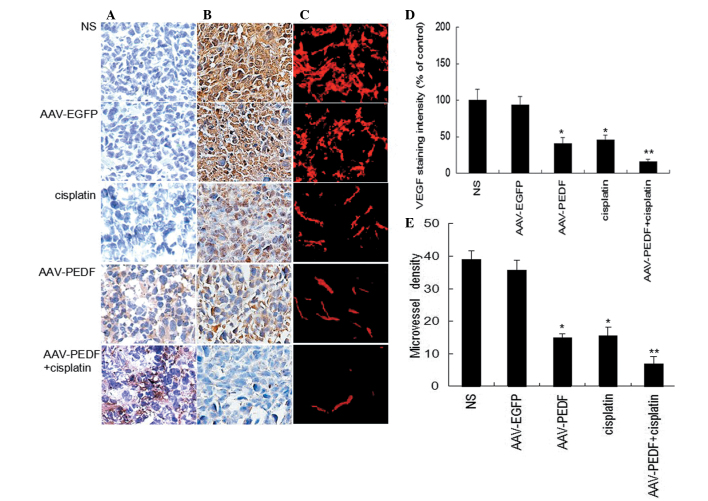
The combination of adeno-associated virus (AAV)-human pigment epithelium-derived factor (hPEDF) and cisplatin inhibited tumor angiogenesis *in vivo*. (A) hPEDF expression in vivo was analyzed by immunohistochemical staining (magnification ×400). (B) Immunohistochemical staining of vascular endothelial growth factor (VEGF) (magnification ×400) and the staining intensity of VEGF was quantified (D). (C) The combination of AAV-hPEDF and cisplatin greatly inhibited tumor angiogenesis in the LLC tumor (magnification ×400). (E) The average microvessel number in the tumors of the combined group was markedly decreased. ^*^P<0.05 and ^**^P<0.01 compared with normal saline (NS) or AAV-enhanced green fluorescent protein (EGFP).

hPEDF expression was investigated in tumor sections from each group by immunohistochemistry. hPEDF staining was strongly positive in the tumor tissue in the group treated with AAV-hPEDF alone and the group treated with a combination of AAV-hPEDF plus cisplatin. Compared with the AAV-hPEDF group, there was stronger hPEDF staining in the group treated with a combination of AAV-hPEDF and cisplatin (Fig. 3A).

These results demonstrate that the combined use of cisplatin and AAV-hPEDF markedly inhibited tumor growth and prolonged the survival time in C57BL/6 mice.

### Enhanced effect of AAV-hPEDF and cisplatin combination treatment on suppression of tumor angiogenesis and induction of tumor apoptosis in vivo

To analyze whether the inhibitory effect of the combination therapy on tumor growth was associated with the suppression of tumor angiogenesis, tumor tissues from each group were immunostained for CD31 and MVD. MVD expression was markedly lower in the combination treatment group compared with the groups treated with cisplatin or AAV-hPEDF alone (Fig. 3C and E, P<0.05) as well as with the two control groups (Fig. 3C and E, P<0.01). VEGF expression in the cisplatin, AAV-hPEDF, and combination groups was lower than that in the control groups, and VEGF expression in the cisplatin plus AAV-hPEDF group was lower than in either of the groups treated with cisplatin or AAV-hPEDF alone (Fig. 3B and D). These data demonstrate that combination treatment significantly inhibits tumor angiogenesis *in vivo*.

**Figure 4:**
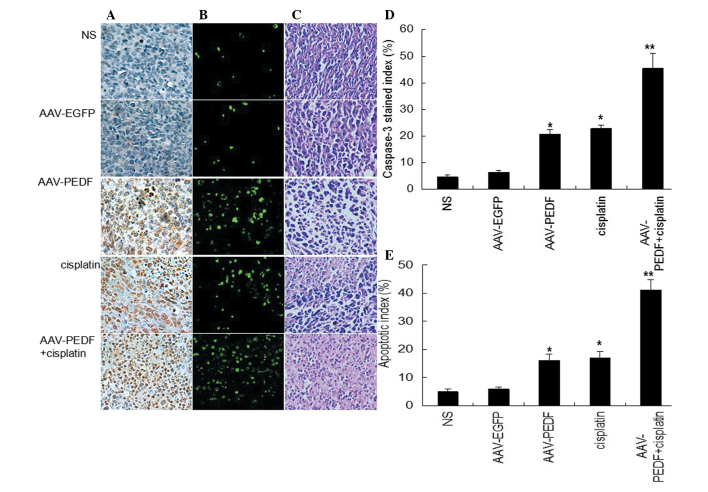
The combination of adeno-associated virus (AAV)-human pigment epithelium-derived factor (hPEDF) and cisplatin induced tumor apoptosis *in vivo*. (A) Immunohistochemical staining of caspase-3 in Lewis lung carcinoma (LLC) tumors (×400). The average caspase-3 stained index was calculated for each group (D). (B) Apoptotic cells were identified by transferase-mediated dUTP nick end labelling assay in LLC tumor sections (×400). The mean apoptotic index was calculated (E). (D) Hematoxylin and eosin staining of LLC tumors in each group (×400). ^*^P<0.05 and ^**^P<0.01 compared with normal saline (NS) or AAV-enhanced green fluorescent protein (EGFP).

Treatment-induced tumor apoptosis was detected by a TUNEL assay. Apoptotic cells were present at low levels in tumors from the NS-treated and AAV-EGFP-treated groups, whereas cisplatin or AAV-hPEDF treatment markedly induced apoptosis in tumor cells. However, the apoptosis rate in the combination treatment group was markedly higher than that in the cisplatin- or AAV-hPEDF-treated groups (Fig. 4B). Furthermore, the activated cell death form of caspase-3 was assessed by immunohistochemistry analysis. The average caspase-3 stained index in the cisplatin- or AAV-hPEDF-treated groups were similar (Fig. 4A and D). The combined therapy resulted in a marked increase in the caspase-3 index, exceeding those of the single treatments (Fig. 4A and D, P<0.05). Additionally, in hematoxylin and eosin staining of tumors, larger areas of tumor necrosis were observed in the cisplatin plus AAV-hPEDF group (Fig. 4C).

### Reduction of body weight loss caused by cisplatin after AAV-hPEDF treatment in mice

Notably, the body weight of mice treated with cisplatin alone (2 mg/kg intraperitoneally every three days) was found to be reduced by 20.6, 22.3 and 21.2% during the experimental period, compared with the NS-, AAV-EGFP-, and AAV-hPEDF-treated mice, respectively (Fig. 2D), whereas that of mice in the combined therapy group did not change (Fig. 2D). In addition, the average net body weight of the animals was calculated to remove the impact of tumor weight on body weight. The net body weight of cisplatin monotherapy-treated animals demonstrated a marked reduction compared with the other four groups (Fig. 2E, P<0.05). These findings indicate that AAV-hPEDF may markedly decrease cisplatin-induced side effects in mice.

## Discussion

Cisplatin is an attractive anticancer agent, frequently used for treatment of multiple human cancers, including ovarian, head and neck, liver and lung cancer, and other solid tumors. The usefulness of cisplatin is limited by its toxicity to nonmalignant tissues or organs, and by intrinsic and acquired resistance to the drug (1,2). Therefore, novel therapeutic approaches are required to decrease the drug dosage, diminish side effects and enhance the therapeutic efficacy to achieve successful use of cisplatin in cancer treatment. Certain studies have reported that supplementing conventional treatment with gene therapy may have synergistic or enhanced efficacy on inhibiting tumor growth (3,5), as gene therapy and chemotherapy act by different mechanisms. Recombinant vectors based on AAV are safe vehicles with potential for gene transfer and gene therapy (6). In the present study, cisplatin was combined with AAV-mediated hPEDF gene to investigate the effect of this combined treatment on tumor growth.

Although initially labeled as a neurotrophic factor, PEDF was later identified to have potent antivascular activity (13), with a demonstrated ability to suppress the growth of various malignancies *in vivo* (22–24). As a potent endogenous inhibitor of neovascularization, PEDF appears to inhibit pathological vessel formation without altering native vasculature (25), and is nontoxic and stable when applied by virus-mediated gene transfer (23,24). The virus (adenovirus and AAV)-meditated PEDF gene has been intensively investigated in the treatment of various types of cancers (22–24). However, it is difficult to eradicate cancer cells using AAV-PEDF alone. Thus, a combined approach of AAV-PEDF and traditional cytotoxic drugs may be beneficial in eradicating lung cancer cells. In the present study, the results suggest that AAV-PEDF conjugated to cisplatin efficiently expressed the PEDF gene in the target cells; this expression was significantly increased by cisplatin treatment and the combination treatment resulted in decreased angiogenesis *in vitro* by inhibiting tube formation (Fig. 1). The *in vivo* experiments in the present study indicated that the combination therapy of AAV-PEDF and cisplatin inhibited tumor growth more efficiently, prolonged survival time, resulted in greater suppression of tumor angiogenesis and exhibited more marked induction of tumor apoptosis *in vivo*, than either treatment alone. In addition, the combination therapy protected tumor-inoculated mice from cisplatin-induced body weight loss (Fig. 2). Therefore, the combinational strategy of AAV-PEDF and cisplatin has potential for use in clinical lung cancer therapy.

The results indicate that AAV-PEDF and low-dose cisplatin may each potentiate the anticancer properties of the other; however the molecular mechanism responsible for the interaction between AAV-PEDF and low-dose chemotherapy remains unclear. AAV-PEDF exerts its antitumor effects through overexpression of PEDF mediated by the AAV vector. PEDF overexpression not only results in a decrease of tumor microvessel density and downregulation of VEGF expression, but also an increase of tumor cell apoptosis, which has both an indirect and direct effect on tumors (14). Conversely, as a cytotoxic agent, cisplatin interferes with DNA synthesis and causes DNA cross-linking that modulates cell cycle progression, thus ultimately inducing tumor cell apoptosis (26). Additionally, previous studies have shown that AAV vector-mediated infection, together with irradiation, UV light and various chemotherapeutic agents, enhances transgene expression (19,20). In the present study, it was demonstrated that low-dose cisplatin was capable of increasing AAV infection in murine LLC cells using AAV-EGFP viral particles. The infection efficiency of the AAV vector harboring hPEDF was similar to that of AAV-EGFP, as predicted. In animal experiments, cisplatin was also revealed to enhance AAV vector-mediated PEDF expression. These effects may contribute to the synergistic suppression of tumor growth. However, the precise antitumor mechanism of the combination treatment requires further investigation.

Although cisplatin is effective against certain tumors, its severe side effects in normal tissues and organs limits its application in cancer therapy. In the present study, intraperitoneal injection of cisplatin resulted in an apparent decrease in body weight; however, AAV-hPEDF was observed to protect tumor-inoculated mice from cisplatin-induced body weight loss (Fig. 2D). Furthermore, the net body weight of the combined therapy group was markedly higher than that of the cisplatin only-treated group (Fig. 2E). However, whether AAV-hPEDF protects against the nephrotoxicity caused by cisplatin requires further investigation.

In conclusion, to the best of our knowledge, the present study is the first to combine AAV-mediated PEDF and low concentrations of cisplatin for cancer therapy. The combined treatment of AAV-hPEDF and cisplatin markedly prolonged the survival time of the mice and suppressed tumor growth, and also protected against cisplatin-related toxicity. These findings suggest that combination of AAV-hPEDF and cisplatin has potential as a novel therapeutic strategy for human lung cancer and other solid tumors.

## Abbreviations

PEDF: pigment epithelium-derived factor
AAV: adeno-associated virus
MVD: microvessel density
LLC: lewis lung carcinoma
VEGF: vascular endothelial growth factor
HUVECs: human umbilical vein endothelial cells
NS: normal saline
